# Activating transcription factor 3 inhibits NF‑κB p65 signaling pathway and mediates apoptosis and cell cycle arrest in cervical cancer cells

**DOI:** 10.1186/s13027-022-00475-7

**Published:** 2022-12-15

**Authors:** Amirhossein Akbarpour Arsanjani, Haniyeh Abuei, Abbas Behzad-Behbahani, Zahra Bagheri, Rita Arabsolghar, Ali Farhadi

**Affiliations:** 1 Division of Medical Biotechnology, Department of Medical Laboratory Sciences, School of Paramedical Sciences, Shiraz University of Medical Sciences, Shiraz, Iran; 2 Diagnostic Laboratory Sciences and Technology Research Center, School of Paramedical Sciences, Shiraz University of Medical Sciences, Shiraz, Iran

**Keywords:** ATF3, NF-κB, Ca Ski, HPV16, Cervical cancer

## Abstract

**Background:**

As a novel tumor suppressor mediator, activating transcription factor 3 (ATF3) has recently aroused an interest in its possible therapeutic applications in various cancers. In this study, we evaluated the effect of ATF3 overexpression on the cellular level of nuclear factor kappa B (NF-κB) in human papillomavirus (HPV)-infected Ca Ski cells. Further, we examined whether ATF3 could mediate cell cycle arrest and alter the apoptosis level of Ca Ski cells.

**Methods:**

The biological behavior of Ca Ski cells was evaluated prior and subsequent to the overexpression of ATF3 by MTT assay, fluorescence microscopy, cell cycle and annexin V/PI flow cytometric analysis. The effect of ectopic ATF3 expression on the cellular level of NF-κB in HPV-positive cells was evaluated by western blotting assay.

**Results:**

The overexpression of ATF3 in Ca Ski cells led to significant apoptosis and cell cycle arrest in the G1 phase. Western blotting assay revealed a discernible reduction of NF-κB p65 level in cervical cancer cells.

**Conclusion:**

ATF3 acts as a tumor suppressor factor in HPV16-infected Ca Ski cells and exerts anti-cancer effects on HPV16-related cervical cancer cells potentially by hindering cell growth and inducing cell cycle arrest through the down-regulation of NF-κB. Our results suggest that ATF3 induction or NF-κB suppression may be useful targets for HPV16-related cervical cancer prevention and treatment.

**Supplementary Information:**

The online version contains supplementary material available at 10.1186/s13027-022-00475-7.

## Introduction

Cervical cancer has been reported as the fourth most common gynecological cancer and accounted for approximately 604,000 new cases and about 342,000 deaths worldwide in the year 2020 [1]. Persistent infection with oncogenic human papillomaviruses (HPVs) is the necessary cause of cervical cancer [2]. High-risk HPVs such as HPV16 and 18 are associated with cervical cancer lesions. In the event of carcinogenesis, HPV16 accounts for ~ 53% of cases [2]. HPV takes advantage of p53 degradation through E6 viral oncoprotein for its own replication and integration into the host genome. Moreover, the degradation of pRb by E7 oncoprotein causes unplanned entry of the infected cells into the S phase of the cell cycle which eventually promotes cell proliferation [3].

Primary methods for the treatment of cervical cancer including surgery, chemotherapy, and radiotherapy prevent tumor growth, prolong lifespan, and decrease the possibility of recurrence [4]; however, only up to one-third of patients with metastatic and recurrent types of the disease respond to these common treatments, and the responses are short-lived and limited to a few months [5]. Over the last few years, many studies have aroused an interest in activating transcription factor 3 (ATF3) as a novel tumor suppressor mediator in glioblastoma, as well as colon, lung, bladder, and cervical cancer cells and its possible therapeutic applications [6–10]. As a member of the ATF/cAMP response element-binding (CREB) protein subfamily, ATF3 is a stress-inducible transcriptional factor [11]. Besides, ATF3 is a basic region leucine zipper transcription factor and can bind to specific regions on DNA or form dimers with some factors or intracellular proteins such as AP-1, C/EBP, NF-κB etc. [12]. Studies have shown that ATF3 binds to the p53 factor in HPV-negative cells and prevents its ubiquitination and degradation by intracellular MDM2 ubiquitin ligase [13–16]. On the other hand, ATF3 expression is often decreased in HPV-infected cervical cells, which is consistent with the microarray and semi-quantitative reverse transcription polymerase chain reaction (RT-PCR) data showing a marked reduction of ATF3 expression in E6-expressing HOK/Bmi-1 cells [17, 18]. In addition, a previous study has reported that ATF3 competes with E6-associated protein (E6AP) ubiquitin ligase to bind to E6 in cervical cancer cells, thereby acting as a suppressor of the E6 viral protein [7].

Nuclear factor kappa B (NF-κB) has recently received a great deal of attention for its role in the onset and progression of human cancers as well as resistance to cancer treatment. The NF-κB family is comprised of transcription factors that play a fundamental and complex role in innate immunity, inflammation, viral replication, and cancer. Five members of this protein have been identified in mammals: p65 (RelA), RelB, c-Rel, NF-κB 1 (p105/p50), and NF-κB 2 (p100/p52) [19]. All members can form different types of homo- or heterodimers that are necessary for their activation and translocation to the nucleus [20]. The classic NF-κB form is a heterodimer of p50 and p65 subunits. NF-κB is normally held in an inactive form in the cytosol by inhibitor of κB (IκB) proteins. Thus, NF-κB activation can occur within minutes through release from IκB [21]. Similar to ATF3, NF-κB has a bilateral role in cancer. On the one hand, NF-κB activation is a part of the acute inflammatory immune response which comprises high activity of cytotoxic immune cells to target transformed cells [22]. On the other hand, NF-κB is constitutively activated in many cancers and can perform various pro-tumorigenic functions [23]. NF-κB activation by HPV infection plays a pivotal role in the innate and adaptive immune response of the host. The virus causes the down-regulation of NF-κB to diminish the inhibitory effect of the immune response on its replication resulting in a status of persistent HPV infection [24]. NF-κB becomes constitutively activated again during the progression to high-grade intraepithelial neoplasia and cervical cancer [25]. A recent study has reported that ATF3 can reduce NF-κB activity through interaction with the p65 subunit to form a complex containing histone deacetylase1 (HDAC1) and binding to NF-κB-specific binding sites on the target gene promoter. This reduces the expression of the target genes by altering the properties of the promoter. In addition, HDAC1 can help the removal of NF-κB from the nucleus through the deacetylation of p65 [26].

In our previous study, we demonstrated that ATF3 acts as a tumor suppressor factor in HPV18-related cervical cancer cells which mediates apoptotic functions through a p53-independent pathway [27]. However, it was unknown whether ATF3 stimulates or represses tumorigenesis in HPV16-infected cells. Furthermore, the effect of ATF3 overexpression on the cellular level of NF-κB p65 in such cancerous cells is unknown. Herein, we overexpress the ATF3 gene in HPV16-infected Ca Ski cells and demonstrate that this leads to a significant cell cycle arrest in the G1 phase. In addition, we show that ATF3 overexpression results in a significant reduction of NF-κB level in Ca Ski cells.

## Materials and methods

### ATF3 gene cloning in pCMV6-GFP vector

The amplification and insertion of ATF3 into the pCMV6-AC-IRES-GFP vector and the construction of GFP-expressing pCMV6-ATF3 recombinant plasmid were performed according to the previously described method [27]. After transformation into *E. coli* DH5α competent cells purchased from the national cell bank of Iran (Pasteur Institute, Tehran, Iran), enzyme digestion, colony PCR, and Sanger sequencing were used to validate the recombinant plasmid. The amplified plasmids in *E. coli* DH5α cells were purified by GF1 Nucleic Acid extraction kit (Vivantis Technologies Co. Malaysia) according to the manufacturer’s protocol.

### Cell culture and transfection

Ca Ski cells were purchased from the American Type Culture Collection (ATCC; Manassas, VA) and cultured in a complete growth medium containing Dulbecco’s modified Eagle’s medium (DMEM), 10% Fetal Bovine Serum (FBS), and 1% Pen/Strep (all Sigma-Aldrich, St. Louis, MO, USA), and incubated in 5% CO2 and 37 °C. The transfection was carried out according to the standard protocol [28, 29]. One day prior to the transfection, Ca Ski cells were trypsinised and seeded in cell culture plates with 7.5 × 10^5^ cells per well in a 6-well dish. Once cells were grown to the confluence of 60–70%, the medium was replaced with fresh DMEM medium 2 h before transfection. 3–7 µg of plasmid was mixed with 25 µL of CaCl2 (2.5 M) solution. The dispersion was incubated for 5 min. Subsequently, the volume of the dispersion was adjusted to 400 µL with distilled H_2_O. Then, 200 µL of 2× HEPES-buffered saline (280 mM NaCl, 10 mM KCl, 1.5 mM Na_2_HPO_4_, 12 mM glucose, 50 mM HEPES pH 7.05) was added to the dispersion drop-wise while the above mixture was being gently mixed. The mixture was incubated at room temperature for 16 min and then added to the Ca Ski cells. The cells were incubated in the calcium phosphate-containing medium for 16 h and subsequently washed with PBS three times. Finally, Ca Ski cells were cultured in fresh complete growth medium for 48 h.

### Fluorescence microscopy and flow cytometric analysis

The efficiency of transfection was evaluated by fluorescence microscopy and transmission light microscopy. Transfected cells were examined under a 20-fold objective lens microscope (Olympus, Tokyo, Japan) 24, 48, and 72 h post-transfection. Fluorescent images were characterized through the GFP filter cube. Transfection efficiency was determined by counting total cells and green ones. Four images were taken at each time point and evaluated individually. For flow cytometric analysis, the cells were washed with PBS 24, 48 and 72 h post-transfection and subsequently detached from the cell culture plate with trypsin-EDTA solution. Cells were centrifugated at 350 g for 5 min, resuspended in PBS, and examined for fluorescent intensity with a FACSCalibur™ flow cytometry system (BD Biosciences; San Jose, CA, USA). In each experiment, a total of 20,000 cells were considered for analysis and the data were analyzed using FlowJo software version 10.0 (FlowJo LLC, Ashland, OR, USA).

### Cytotoxicity assay

In order to determine the cytotoxicity of ATF3, MTT assay was performed. Briefly, cells were seeded into 96-well plates at the density of 7000 cells per well in 200 µL DMEM medium and incubated at 37‎°C‎ and ‎5% CO2. Once Ca Ski cells were grown to the confluence of 70%, they were incubated with calcium phosphate-precipitated pCMV6-ATF3 and mock plasmid with concentrations ranging between 0.1 and 1 µg according to the above-mentioned transfection protocol. Cell viability was determined 24, 48 and 72 h post-transfection by adding fresh medium containing 20 µL MTT solution (5 mg/mL) (Sigma-Aldrich), followed by a four-hour incubation for crystal formation. Next, ‎150 µL of DMSO (Sigma-Aldrich) was added‎ to each well. Finally, the absorbance was measured at the wavelength of 570 nm with a Stat Fax 2100 microplate reader (Awareness Technology Inc., Palm City, FL, USA). ‎The MTT assay was performed in triplicate.

### Cell cycle analysis

Ca Ski cells were seeded in a 24-well plate at a density of 7 × 10^4^ cells per well with 0.5 mL of medium in each well. Forty-eight hours after transfection with 5 and 7 µg of pCMV6-ATF3 and 7 µg of mock plasmid, the harvested cells were washed with warm PBS and fixed with cold absolute ethanol (with the final concentration of EtOH being 70%) at 4 °C for 1 h. In order to remove the ethanol, the fixed cells were centrifuged at 4,500 rpm for 10 min. Then, the cells were washed with warm PBS twice and stained with 0.5 mL of warm PI solution (consisting of 0.35 mL PI stock solution (1 mg/mL), 0.7 mL RNase A stock solution (1 mg/mL), and 6 mL PBS, all of which were purchased from Sigma-Aldrich), and incubated at room temperature in a dark place for 30 min. Twenty-thousand cells were considered for flow cytometric analysis in each group. Cell cycle analysis was carried out using a flow cytometer and the data were analyzed with FlowJo software version 10.0.

### Cell lysis and protein assay

Ca Ski cells were seeded in 6-well cell culture plates with each well containing 1.5 mL medium. Cells were incubated at 37‎ °C‎ and ‎5% CO2 overnight and subsequently transfected as described above. Forty-eight h after transfection, cells were harvested and washed twice with cold PBS. Then, the cell suspension was sonicated (Amplitude: 80–100, 2 × 30 s, with 30-s cooling intervals) in RIPA buffer (1% Triton X100, 0.1% SDS, 150 mM NaCl, 50 mM Tris-HCl, pH 8.0) supplemented with 25 µL protease inhibitor cocktail (Sigma-Aldrich). Samples were centrifugated at 15,000 g for 20 min at 4 ℃ and the supernatants were collected. Protein concentrations of both test and control cells were determined by means of Bradford protein assay (Bio-Rad Bradford protein assay) and an absorbance standard curve was generated.

### NF-κB and ATF3 western blotting

A 5× sample buffer (0.25 M Tris-HCl pH 6.8, 50% glycerol, 10% SDS, 0.1% bromophenol blue, 5% β-mercaptoethanol) was added to the cell lysate. The mix was immediately boiled at 95 °C for 10 min. Next, electrophoresis was performed in a 12%-SDS-polyacrylamide gel for equal amounts of proteins from each sample. Proteins were then transferred onto polyvinylidene fluoride (PVDF) membranes (Millipore, Feltham, United Kingdom) using Bio-Rad Mini Trans-Blot® electrophoretic transfer cell at 300 mA for 3 h. Membranes were incubated in a blocking solution (5% skim milk, 0.05% Tween 20) for 16 h and then washed with TBST buffer (150 mM NaCl, 50 mM Tris-base, and 0.05% Tween 20) for 15 min. Afterwards, the quantities of NF-κB and ATF3 protein were measured using monoclonal anti-NF-κB (p65) and anti-ATF3 antibodies (1:500, Santa Cruz Biotechnologies, Santa Cruz, CA, USA), respectively. β-actin (dilution, 1:5000, Santa Cruz Biotechnology) served as an internal control. Following washing with TBST for three times, the membrane was incubated with horseradish peroxidase-conjugated secondary antibody (1:10000, Sigma-Aldrich) at room temperature for 1 h and then washed with TBS buffer twice (50 mM Tris-base and 150 mM NaCl). Finally, 10 mL of 3,3′-Diaminobenzidine (DAB) (Sigma-Aldrich) substrate (5 mg of DAB in 10 mL DDW and 5 µl H_2_O_2_) was added to the PVDF surface. DDW was used to stop the reaction.

### Apoptosis assay

The level of apoptosis was assessed in 2 × 10^5^ transfected Ca Ski cells. Cells were transfected as described above and washed with 1 mL of PBS. After treatment with 250 µL of trypsin solution, cells were collected by centrifugation at 350 g for 5 min. The supernatant was discarded and cells were washed twice in cold PBS. Afterwards, apoptosis assay was carried out using a PE-Annexin V Apoptosis Detection Kit (BD Biosciences, Bedford, MA, USA) based on the manufacturer’s instructions. Ca Ski cells transfected with PCMV6-ATF3 and mock plasmid along with untreated cells were incubated with 5 µL Annexin V (conjugated with PE) after resuspension with 400 µL binding buffer (10X binding buffer: 0.1 M HEPES/NaOH (pH 7.4), 1.4 M NaCl, 25 mM CaCl2). Briefly, after 15 min of incubation at room temperature in the dark, cells were stained with 5 µL 7-AAD and incubated for another 15 min prior to flow cytometric analysis. A FACSCalibur™ flow cytometer (BD Bioscience, San Jose, CA, USA) was used to analyze the cells and further investigation was carried out with FlowJo software version 10.0.

### Statistical analysis

GraphPad Prism software version 8.0 (GraphPad Software Inc., La Jolla, CA, USA) was used for all analyses. Data were presented as mean ± SD and *p*-values < 0.05 were considered statistically significant.

## Results

### ATF3 gene cloning in pCMV6 vector

**Fig. 1:**
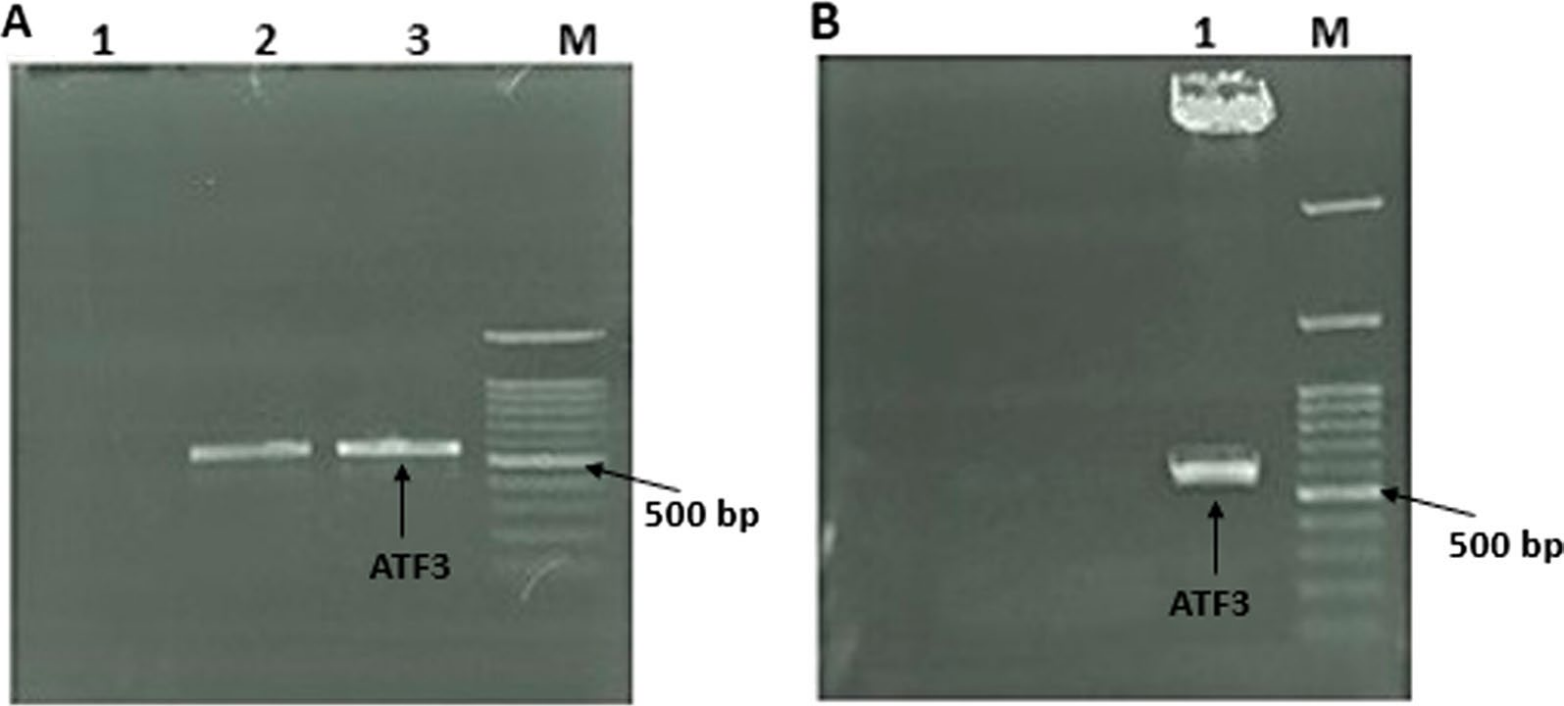
**A** Colony PCR of ATF3, Lane M: ‎Size marker (100–3000 bp), Lane 1: NTC. ‎Lane 2 and 3: ATF3 (553 bp).‎ **B** Double digestion of pCMV6-ATF3, Lane M: ‎Size marker (100–3000 bp), Lane 1: digested pCMV6-AC-IRES-GFP plasmid (6740 bp) and ATF3 (553 bp)

The integration of ATF3 into the pCMV6-AC-IRES-GFP plasmid was confirmed by the amplification of the ATF3 gene by PCR. Agarose gel electrophoresis visualized a ~ 553-bp band suggesting the successful cloning of the ATF3 segment (Fig. 1A). In the next step, double digestion of the pCMV6-ATF3 recombinant plasmid revealed corresponding bands of about 553 and 6740 bp (Fig. 1B), confirming the fidelity of the cloning. Finally, the cloning was confirmed by Sanger sequencing.

### Transfection of recombinant vector into ca. ski cells

**Fig. 2:**
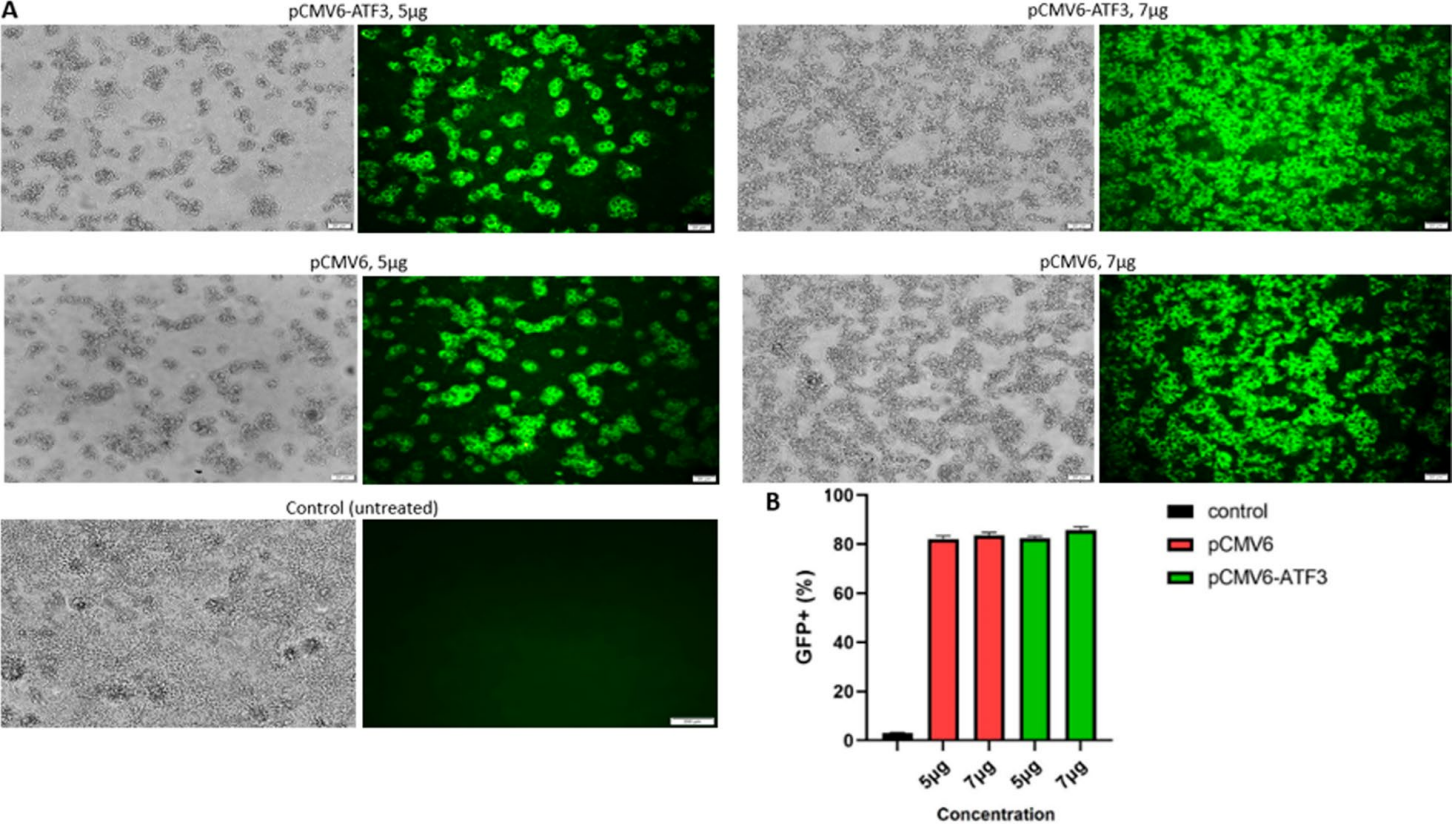
Fluorescent intensity for pCMV6-ATF3, mock-transfected and control group using fluorescence microscope and Flow Cytometer. **A** The fluorescence microscope results showed that the percentage of transfected cells with pCMV6-ATF3 and pCMV6 was more than 80%. (Zoom in: 20 μm). **B** The flow cytometry analysis showed that the average transfection efficiency for 7 µg pCMV6-ATF3 and mock-transfected cells after 48 h was 85.67% and 83.56%, respectively (*p* < 0.005)

Increasing the amount of DNA, we observed a rise in the fluorescent intensity of the cells. However, the percentage of the transfected cells did not change when more than 7 µg DNA was used for transfection. Moreover, fluorescent intensity reached its maximum value 48 h after transfection. Accordingly, the experimental conditions were designed as follows: 5 and 7 µg of DNA were used for transfection and the cells were analyzed 48 h post-transfection. Finally, images of the transfected cells were captured with a fluorescent microscope and analyzed manually to quantify the efficiency of transfection. Transfected and total cells were counted and the transfection efficiency was calculated according to the ratio of the former to the latter. The percentage of the cells transfected with pCMV6-ATF3 was found to be more than 80%. Furthermore, mock-transfected Ca Ski cells revealed transfection efficiency of over 80%. Using flow cytometry analysis, the average transfection efficiency was up to 85.67% and 83.56% for 7 µg pCMV6-ATF3 and 7 µg pCMV6, respectively at 48 h post-transfection (Fig. 2).

### MTT ‎assay

**Fig. 3:**
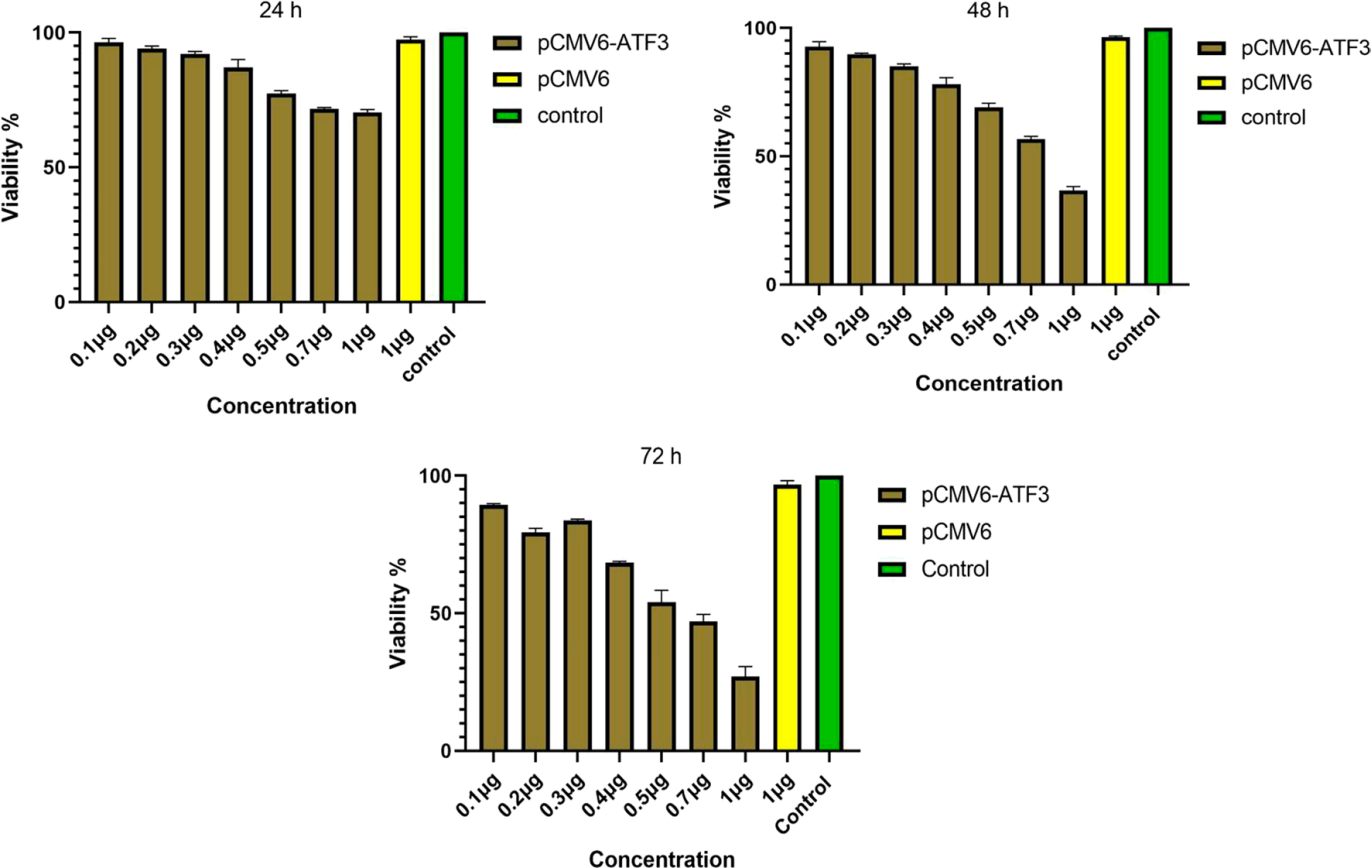
Ca Ski cells were transfected with pCMV6-ATF3 plasmid at the concentrations of 0.1, 0.2, 0.3, 0.4, 0.5, 0.7, and 1 µg, and their viability was evaluated by MTT assay after 24, 48 and 72 h. The results show a statistically significant reduction in the viability of Ca Ski cells when transfected with pCMV6-ATF3 plasmid compared with untreated groups at all conditions (*p* < 0.05), but no significant reduction in the viability of mock-transfected groups (*p* > 0.05)

MTT assay was used to determine the viability of Ca Ski cells after transfection with pCMV6-ATF3. Cells were treated with pCMV6-ATF3 at a series of DNA concentrations (0.1-1 µg) and mock (1 µg) for 24, 48 and 72 h. MTT assay showed that ATF3 overexpression at the concentration of 1 µg resulted in the maximum inhibition of Ca Ski cell growth (73% at 72 h) which was significantly higher than untreated cells and mock-transfected ones (*p* < 0.005) as shown in Fig. 3. More details of the numerical values of the MTT assay are presented in Additional file 1: Table S1.

### Cell cycle analysis

**Fig. 4:**
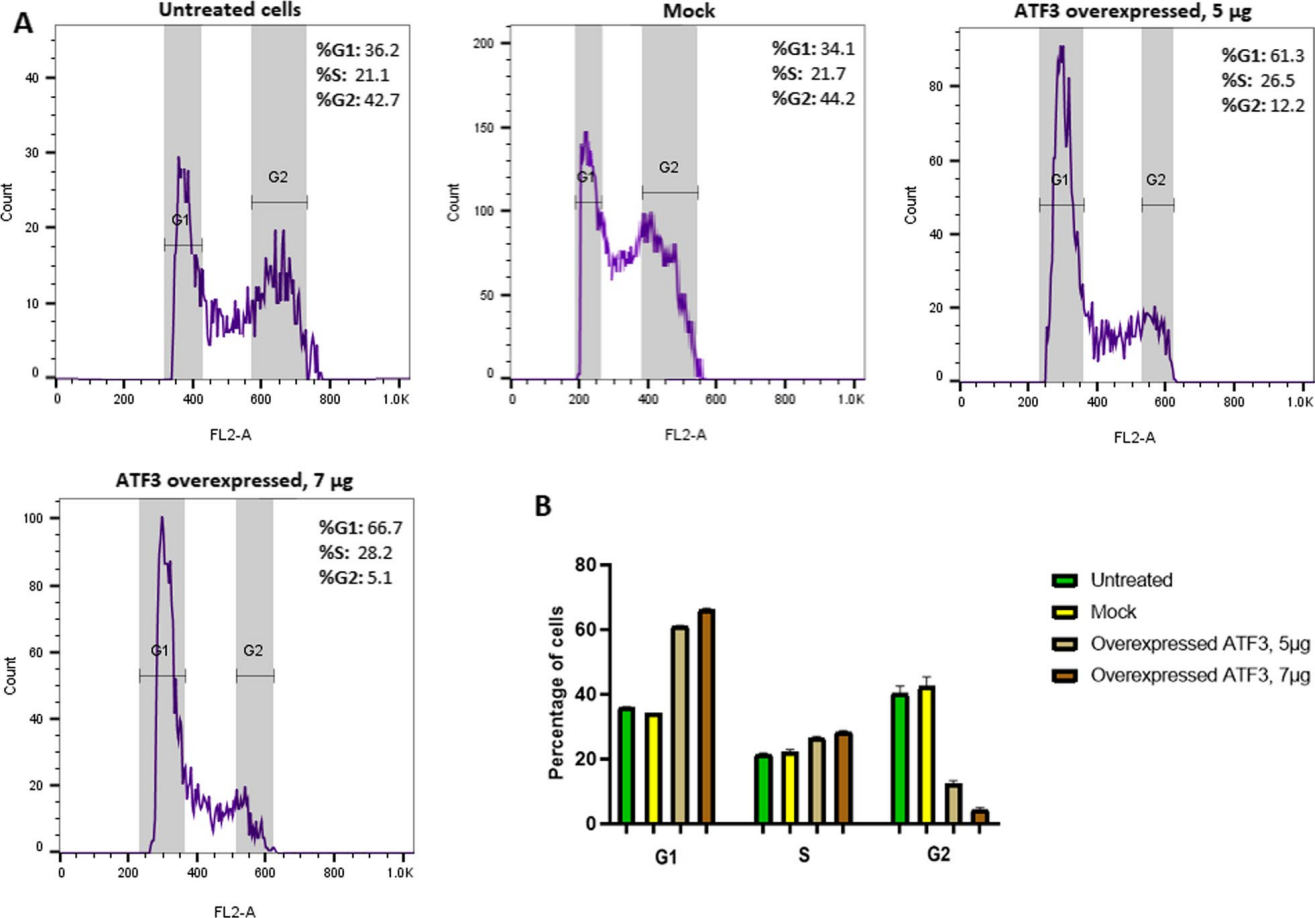
Cell cycle analysis results. **A** Cell cycle histogram represents the percentage of the cells in G1, S and G2/M phases after 48 h compared with mock and control (*p* < 0.0001). **B** The percentage of the cells in G1, S and G2/M phases after 48. No significant difference was found between mock and control groups (*p* > 0.05)

Flow cytometric analysis showed an increase in the percentage of the cells in G1 and S phases following ATF3 overexpression. Conversely, the proportion of the cells in G2/M phase was shown to have decreased. The percentage of the cells in G1 phase was 61.3% and 66.7% in the case of 5 and 7 µg pCMV6-ATF3-transfected cells, respectively (Fig. 4). Our results indicated that pCMV6-ATF3, in both quantities of 5 and 7 µg, was able to induce cell cycle arrest in G1 phase in Ca Ski cells (*p* < 0.0001). No significant difference was found between the mock and control groups (*p* > 0.05).

### Western blotting

**Fig. 5:**
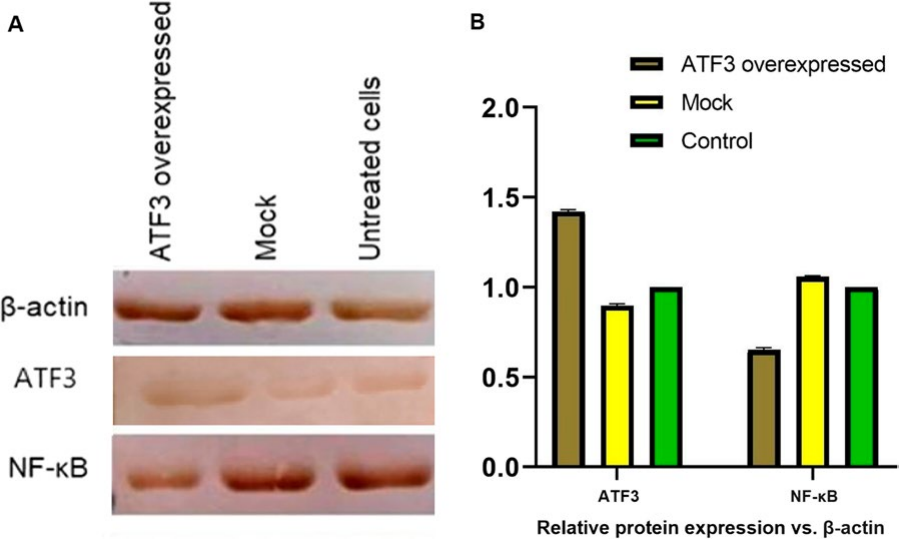
ATF3 and NF-κB protein levels were determined in Ca Ski cells by western blotting. Western blotting was performed on whole-cell lysates using anti-ATF3, anti-NF-κB and anti-β-actin antibodies. **A** pCMV6-ATF3- and mock-transfected Ca Ski cells and untreated ones after 48 h. **B** Densitometry analysis of the relative levels of protein expression after 48 h. pCMV6-ATF3-transfected Ca Ski cells exhibited higher expression of ATF3 compared to the untreated and mock groups (*p* < 0.05). The results of western blotting also demonstrated that the overexpression of ATF3 had a significant effect on NF-κB levels in Ca Ski cells when compared to the untreated and mock-transfected cells (*p* < 0.05)

According to the western blot analysis, the pCMV6-ATF3-transfected cells exhibited a significant increase in the expression of ATF3 protein compared to the untreated and mock groups (*p* < 0.005) (Fig. 5). The results of western blotting also showed that the overexpression of ATF3 had a significant effect on NF-κB levels in Ca Ski cells (*p* < 0.005).

### Apoptosis assay

**Fig. 6:**
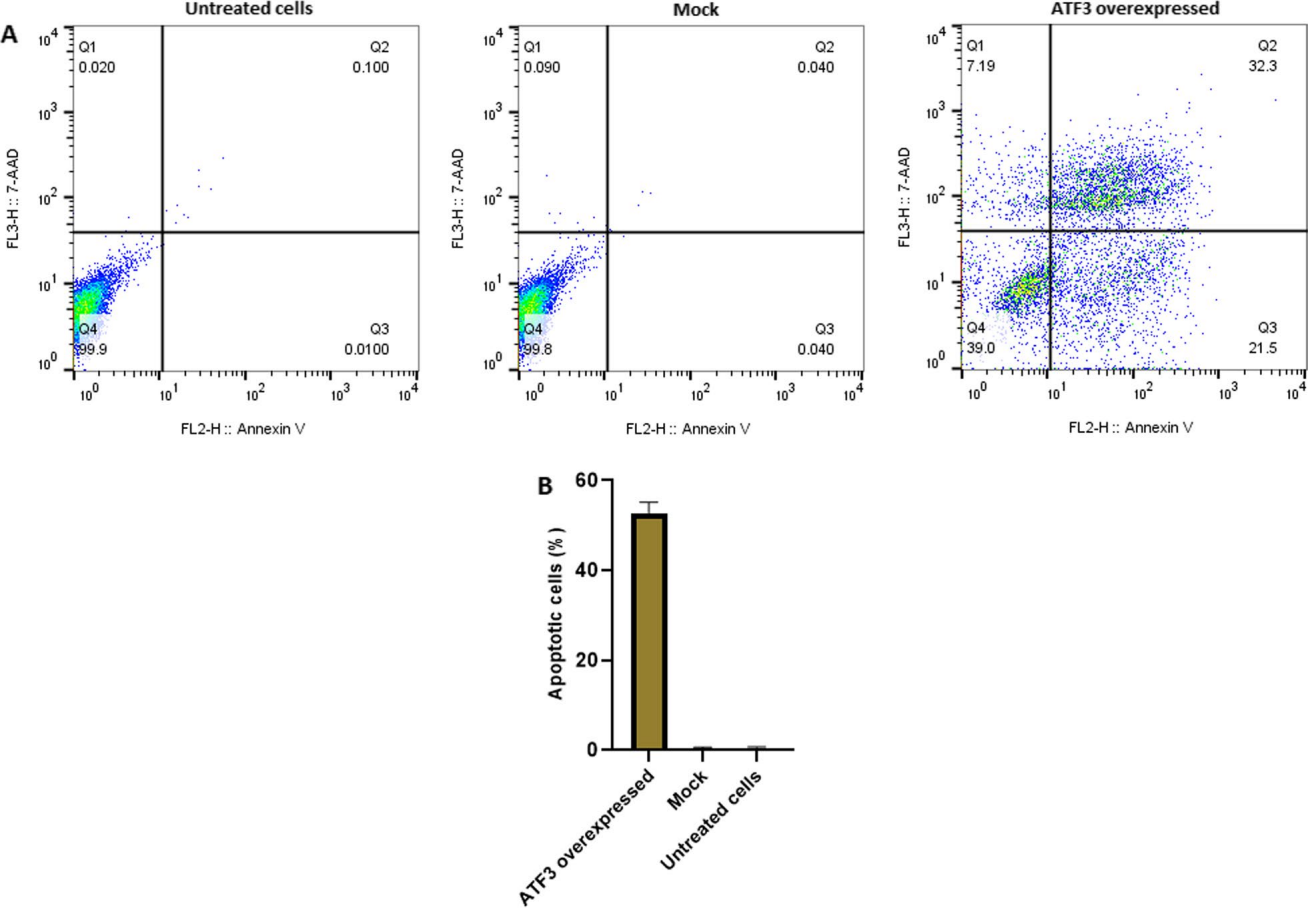
Apoptosis induced by ATF3 overexpression on Ca Ski cells. The histogram represents the percentage of total apoptotic cells in different groups. FlowJo software version 10.0 was used to draw the flow cytometry plots. Ca Ski cells were transfected with 7 µg of pCMV6-ATF3 prior to the measurement of apoptosis level using PE Annexin V apoptosis detection kit after 48 h (**A**). A statistically significant increase in the number of apoptotic Ca Ski cells was observed when compared to the ‎untreated and ‎mock groups (*p* < 0.0001). However, the difference in the number of apoptotic cells between the ‎untreated and ‎mock group was not significant ‎(*p* > 0.4) (**B**). Q1, Q2, Q3, and Q4 indicate necrotic, late apoptotic, early apoptotic, and viable cells, respectively. The percentage of total apoptotic cells was calculated from Q2 + Q3.

The apoptosis induction effect of ATF3 on Ca Ski cells was examined using PE Annexin V apoptosis detection kit. Cells were transfected with pCMV6-ATF3 and mock plasmids for 48 h and then evaluated by flow cytometry. The experimental results shown in Fig. 6 indicate that transfection with pCMV6-ATF3 increases the ‎apoptosis rate up to ‎53.5 ± 1% after 48 h in Ca Ski cells with a significant difference compared to ‎ the untreated and ‎mock groups ‎(*p* < 0.0001).

## Discussion

The investigation of molecular mechanisms in cervical cancer is of great importance for a full understanding of the biology of this highly malignant cancer and the identification of potential therapeutic targets. It has been suggested that ATF3, a principal transcriptional factor, is frequently down-regulated in cervical cancer [21]. However, controversy exists whether ATF3 stimulates or represses tumorigenesis in HPV16-infected cells. Herein, we have demonstrated that ATF3 acts as a tumor suppressor factor which mediates apoptosis and cell cycle arrest in HPV16-related cervical cancer cells. Moreover, for the first time, we have described a novel ATF3-dependent cell cycle arrest induction which impacts on NF-κB expression as one of the most important intracellular transcription factors reported to be constitutively activated in many types of cancers including cervical cancer [23, 24].

We observed that the ectopic overexpression of ATF3 through the transfection of pCMV6-ATF3-GFP vector led to significantly increased apoptosis in Ca Ski cells when compared to the mock and untreated groups. Previous studies have demonstrated that although E6 and E7 oncoproteins disrupt p53 and pRB functioning in HPV16-associated cervical cancers, other apoptosis-related genes may be overexpressed in these patients [30, 31]. For instance, in the absence of functional p53, activated p73 can induce apoptosis as well as the expression of p53 target genes such as Bax or p21 [32]. On the other hand, a former study demonstrated that the ectopic expression of ATF3 in SiHa cells could activate p53, an effect likely achieved through a mechanism that prevents the E6-mediated degradation of the tumor suppressor in HPV16-positive cells. They confirmed this finding by measuring p53 half-life using pulse-chase assays which revealed that the half-life of p53 was significantly increased by the ectopic expression of ATF3 in these cells. In accordance with the mentioned studies, we found that the ectopic expression of ATF3 in Ca Ski cells increased the percentage of Ca Ski cells in the G1 phase of cell cycle which led to cell cycle arrest and apoptotic cell death. While in our previous study, we demonstrated that the percentage of HPV18-infected cells in the G1 phase was 43.2% after optimum transfection with the plasmid expressing ATF3-GFP in 48 h [27], in the present study, it has been revealed and that 66.7% of HPV16-infected cells were arrested in G1 phase which implies that the anticancer effects of ATF3 are dramatically more vigorous in HPV16-infected Ca Ski cells than in HPV18-infected HeLa cells. Apparently, ATF3 induces apoptosis and cell arrest through various pathways in cervical cancer cells based on the type of HPV infection involved. Previously, we showed that in HPV18-infected HeLa cells, ATF3 functioned as a tumor suppressor factor through a p53‑independent pathway which induced apoptosis in the cells with depleted levels of p53 [27]. However, in the case of HPV16 infection, it has been shown that ATF3 competes with E6AP for direct binding to E6, suppresses E6-mediated p53 degradation, and thus restores p53 activity by blocking the recruitment of the ubiquitin ligase to p53, leading to diminished ubiquitination and proteolysis of the latter protein [7]. It may be concluded that ATF3 has a slightly greater tendency to bind to HPV16 E6 compared to HPV18 E6 due to the structural differences between HPV16 E6 and HPV18 E6 proteins, an observation which has been reported earlier [33].

Recent studies indicate that NF-κB pathway plays an important role in the development of cervical cancer [24]. Based on clinicopathological data, it can be concluded that a significant linear relationship exists between the increasing grade of cervical intraepithelial neoplasia (CIN) leading to cervical carcinoma and the intensity of cytoplasmic NF-κB expression which suggests a tumor-promoting role for NF-κB in cervical cancer [34]. In addition, NF-κB plays an important role in the resistance to chemo and radiotherapy. It has been demonstrated that ionizing radiation (IR) can activate NF-κB, and NF-κB activity increases in different cell lines following exposure to cytotoxic agents [35]. Given the important role of NF-κB signaling in the development and progression of tumors and resistance to chemoradiotherapy, targeting NF-κB as a systemic cancer therapy has been investigated extensively [24].

Toll-like receptor 4 signaling pathway, including NF-κB as one of the major molecules of this pathway, is negatively regulated by ATF3. Induced by inflammatory responses, cell death, cytokines, and oxidative stress conditions, it represses inflammation by interacting with the p65 subunit of NF-κB. Previous findings revealed that NF-κB was significantly up-regulated in RAW 264.7 and HEK293 cells transfected with ATF3 siRNA [26]. Therefore, we measured the effect of ATF3 overexpression on the cellular level of NF-κB p65 in Ca Ski cells. A significant reduction of NF-κB level in the Ca Ski cells leading to a significant cell cycle arrest in G1 phase was found. This may imply that ATF3 induces cell cycle arrest by affecting NF-κB-related pathways either directly through binding to the p65 subunit of NF-κB and thus inducing its subsequent attenuation or indirectly by eliciting the regulation of matrix metalloproteinase (MMP) expression and the inhibition of the nuclear translocation of NF-κB in glioblastoma cells as Guenzle et al. reported earlier [36]. In addition, the role of HPV oncoproteins is also of great importance in NF-κB activation which may have a different impact on NF-κB expression in HPV-infected cells. Some studies have shown that HPV16 E6 and E7 regulate NF-κB expression in virus-infected cells [37, 38]. Since it has been observed that ATF3 forms a complex with HPV16 E6 protein as mentioned earlier [7], it can be concluded that ATF3 reduces NF-κB expression through binding to E6 and inhibiting its impact on NF-κB expression. Taken together, ATF3 down-regulates the activity of NF-κB and, as a result, suppresses the tumor in HPV16-infected cells. Therefore, it seems that targeting ATF3 as a potential novel intervention through gene therapy or other therapeutic approaches could be a promising strategy to attenuate NF-κB up-regulation in cervical cancer.

## Conclusion

ATF3 acts as a tumor suppressor factor in HPV16-infected Ca Ski cells and exerts anti-cancer effects on HPV16-related cervical cancer cells potentially by hindering cell growth and inducing cell cycle arrest through the down-regulation of NF-κB. Our results suggest that ATF3 induction or NF-κB suppression may be useful targets for HPV16-related cervical cancer prevention and treatment. Nevertheless, further studies are required to better understand the ATF3-induced pathways of NF-κB down-regulation and cell cycle arrest induction in HPV16-infected cells.

## Supplementary Information

**Additional file 1: Fig. S1.** Ca Ski cells were transfected with pCMV6-ATF3 plasmid and mock at the concentrations of 7 ug and untreated cells after 48 hours. ATF3 and NF-κB protein levels in Ca Ski cells were determined by western blotting. Whole cell lysates were subjected to Western blotting with anti-ATF3, anti-NF-κB and anti- β-actin antibodies.** Table S1.** The numerical values of the MTT assay.

## Data Availability

All data generated during this study are included in this published article.
