# Supplementary material for: Activating transcription factor 3 inhibits NF‑κB p65 signaling pathway and mediates apoptosis and cell cycle arrest in cervical cancer cells
Source: Infect Agent Cancer. 2022 Dec 15;17:62. doi: 10.1186/s13027-022-00475-7 (PMC9753250; doi:10.1186/s13027-022-00475-7)
Supplement: Supplementary file 1 — Additional file 1: Fig. S1. Ca Ski cells were transfected with pCMV6-ATF3 plasmid and mock at the concentrations of 7 ug and untreated cells after 48 hours. ATF3 and NF-κB protein levels in Ca Ski cells were determined by western blotting. Whole cell lysates were subjected to Western blotting with anti-ATF3, anti-NF-κB and anti- β-actin antibodies. Table S1. The numerical values of the MTT assay. [file 13027_2022_475_MOESM1_ESM.pdf]

Supplementary Material

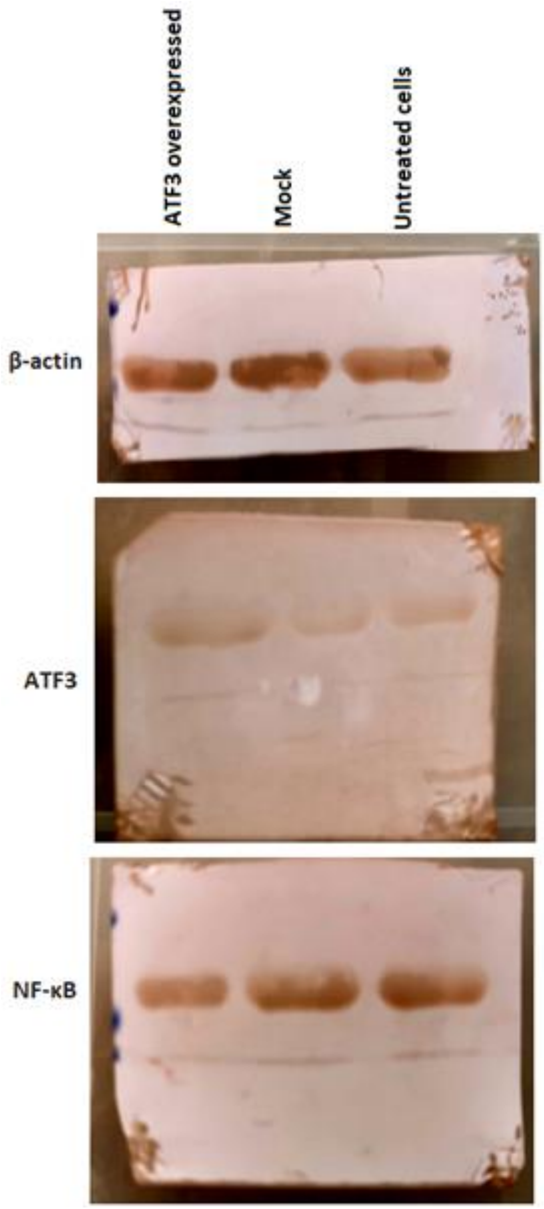

Fig. S1 Ca Ski cells were transfected with pCMV6-ATF3 plasmid and mock at the concentrations of 7 ug and untreated cells after 48 hours. ATF3 and NF-κB protein levels in Ca Ski cells were determined by western blotting. Whole cell lysates were subjected to Western blotting with anti-ATF3, anti-NF-κB and anti- β-actin antibodies

Table S1 The numerical values of the MTT assay

Concentration	Viability % (24 h)	Viability % (48 h)	Viability % (72 h)
0.1 µg pCMV6-ATF3	95	92	89
0.2 µg pCMV6-ATF3	93	89	83
0.3 µg pCMV6-ATF3	91	84	79
0.4 µg pCMV6-ATF3	86	77	68
0.5 µg pCMV6-ATF3	77	68	54
0.7 µg pCMV6-ATF3	72	57	47
1 µg pCMV6-ATF3	70	36	27
1 µg pCMV6	97	96	95
Untreated (control)	99	99	98
